# Characterizing P-glycoprotein and Breast Cancer Resistance Protein interactions of asciminib among other tyrosine kinase inhibitors used in chronic myeloid leukemia

**DOI:** 10.1007/s00280-026-04891-z

**Published:** 2026-05-12

**Authors:** N. E. Verhagen, D. C.W. Touw, J. J.M.W. van den Heuvel, P. H. van den Broek, N. M.A. Blijlevens, J. J.W.M. Janssen, J. B. Koenderink, F. G.M. Russel

**Affiliations:** 1 Department of Pharmacy, Pharmacology and Toxicology, Radboud University Medical Center, Nijmegen, The Netherlands; 2 Department of Hematology, Radboud University Medical center, Nijmegen, The Netherlands

**Keywords:** Breast Cancer Resistance Protein (BCRP), P-glycoprotein (P-gp), Tyrosine kinase inhibitors (TKIs), Chronic myeloid leukemia (CML), Active drug transport

## Abstract

The distribution of tyrosine kinase inhibitors (TKIs) used in the treatment of chronic myeloid leukemia (CML) is influenced by the efflux transporters P-glycoprotein (P-gp) and Breast Cancer Resistance Protein (BCRP). While most TKI-transporter interactions are well established, limited information is available for the novel STAMP inhibitor asciminib, and divergent data exist for the role of P-gp in nilotinib and bosutinib efflux. This study used in vitro cellular and vesicular transport assays to evaluate the substrate and inhibitory profiles of all TKIs currently used in CML treatment. It was confirmed that asciminib is a substrate of both P-gp and BCRP, similar to imatinib and dasatinib, whereas bosutinib was modestly transported by P-gp only. For P-gp, asciminib appeared to be a weak inhibitor, comparable to dasatinib, whereas nilotinib remained the most potent inhibitor. Conversely, asciminib demonstrated notable potency against BCRP, ranking it among the strong inhibitors imatinib, nilotinib, and ponatinib, while dasatinib and bosutinib were less effective. These findings provide further insight into the efflux transporter interactions of asciminib and updated evidence on nilotinib and bosutinib transport by P-gp, with implications for drug resistance and treatment optimization in CML.

**Supplementary Information:**

The online version contains supplementary material available at 10.1007/s00280-026-04891-z.

## Introduction

Chronic myeloid leukemia (CML) is considered functionally curable, as available treatments with tyrosine kinase inhibitors (TKIs) are highly effective, resulting in a life expectancy of patients approaching that of the general population [1]. However, TKI treatment is associated with significant adverse effects, and in a study by Busque et al. it was found that 49.3% of patients switched their first-line (1 L) treatment to second-line (2 L) treatment due to intolerance (65.9% of the 1 L to 2 L switches) or resistance (34.1% of the 1 L to 2 L switches) [2]. Various resistance mechanisms may contribute to this, one of which relates to changes in the cytoplasmatic concentration of TKIs, mediated by cellular influx and efflux transport mechanisms [3]. Because of their lipophilic nature, all CML-associated TKIs can readily cross the cell membrane by passive diffusion [3]. Although, some also claim carrier-mediated uptake of imatinib by the uptake transporter *SLC22A1/*OCT1, while others state that drug influx is solely reliant on passive pH-dependent uptake [4, 5]. Various ATP-binding cassette (ABC) transporters, such as *ABCB1/*P-glycoprotein (P-gp) and *ABCG2/*Breast Cancer Resistance Protein (BCRP), have been identified as (multi)drug resistance proteins involved in TKI efflux [3, 6–9]. Despite this, there is limited in vitro data available regarding asciminib, the most recently approved TKI for CML, particularly with respect to its inhibitory potency against P-gp and BCRP. Furthermore, the involvement of P-gp in the cellular efflux of nilotinib and bosutinib continues to be uncertain due to inconsistent findings. Studies have reported that nilotinib is not transported by P-gp [10, 11], while other investigations found indications for P-gp-mediated nilotinib transport [12–14]. Diverging results have also been published regarding P-gp-mediated bosutinib transport [10, 15–17]. The aim of the current study was to gain more insight into asciminib transport by and inhibitory effect on P-gp and BCRP within the scope of a systematic evaluation of all TKIs approved for CML, including clarifying the unresolved role of P-gp in nilotinib and bosutinib transport. Substrate profiles were assessed using cellular efflux assays, while inhibitory potency was quantitatively determined through membrane vesicle transport assays.

## Methods

### Compounds

Tritium-labelled N-methyl-quinidium chloride ([^3^H]-NMQ) was purchased from SOLVO biotechnology (Szeged, Hungary). Tritium-labelled esterone sulfate ([^3^H]-ES) was purchased from PerkinElmer (Waltham, Massachusetts, United States). Adenosine 5’ monophosphate (AMP), adenosine 5’ triphosphate (ATP), dimethyl sulfoxide (DMSO), elacridar, KO143, magnesium chloride (MgCl_2_), nilotinib, Triton™ X-100 were purchased from Sigma-Aldrich (Zwijndrecht, the Netherlands). Asciminib, bosutinib, dasatinib, imatinib (mesylate), imatinib-d3, nilotinib, ponatinib (hydrochloride) were purchased from Cayman Chemical Company (Ann Arbor, Michigan, United States). Nilotinib-d6 was purchased from Toronto research chemicals (North York, Canada). Tris-Base was from Invitrogen (Waltham, Massachusetts, United States) and sucrose from VWR chemicals (Radnor, Pennsylvania, United States). TS buffer consisted of 25 mM Tris and 625 mM sucrose, pH 7.4. Stop/wash buffer was obtained from PharmTox (www.pharmtox.nl, Nijmegen, the Netherlands). Dulbecco’s modified eagle’s medium (DMEM) was purchased from Thermo Fisher Scientific (Waltham, MA, United States), fetal bovine serum (FBS) was purchased from Greiner Bio-One (Alphen a/d Rijn, the Netherlands). Bio-Rad protein assay dye reagent concentrate was purchased from Bio-Rad (Hercules, California, United States).

### Cell culture

Human embryonic kidney 293 (HEK293) cells were maintained at 37 °C with 5% CO_2_ in DMEM culture medium containing 10% (v/v) FBS. Cells were passaged every 3–4 days when reaching ~ 80% confluency.

### P-gp and BCRP transporter-mediated cellular efflux of TKIs

**Fig. 1:**
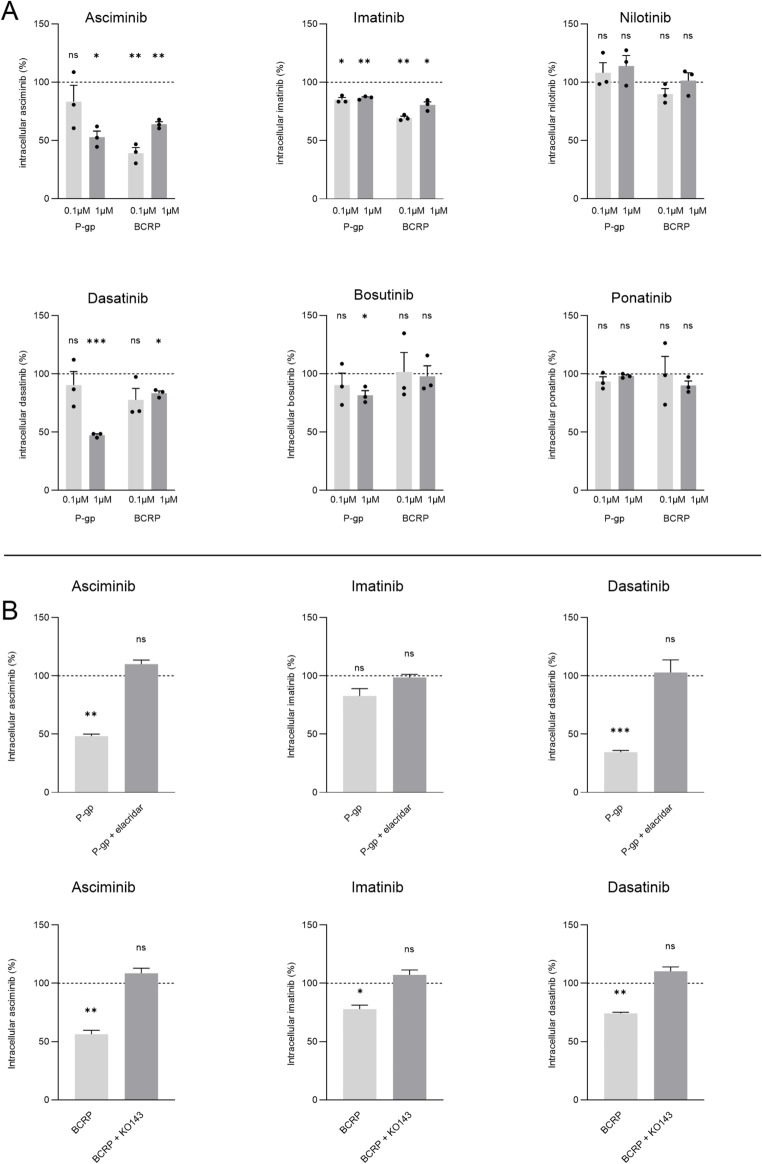
P-gp- and BCRP-mediated transport of asciminib, imatinib, nilotinib, dasatinib, bosutinib and ponatinib was expressed as percentage of the intracellular concentration under control conditions, indicated by the dashed line at 100% (**A**). Light gray bars represent conditions exposed to 0.1 µM TKI, dark gray bars represent conditions exposed to 1 µM TKI. Asciminib, imatinib and dasatinib were further evaluated using elacridar-mediated P-gp inhibition and KO143-mediated BCRP inhibition, to confirm specific transport as percentage of the intracellular concentration under control conditions (**B**). Three separate experiments were performed in triplicate. Data are presented as mean ± SEM. Significance is indicated as *P ≤ 0.05, **P ≤ 0.01, ***P ≤ 0.001, or ‘ns’ for non-significant results.

HEK293 cells were cultured, seeded and transduced with the transporter of interest as described before, using 60 or 90 µL of the recombinant baculovirus [18, 19]. 72 h post-transduction, the culture medium was replaced with medium containing 0.1 µM or 1 µM TKI and incubated for 30 min at 37 °C and 5% CO_2_. Samples were washed and lysed using 50% methanol for LC/MS-MS analysis or 0.1% Triton-X for protein quantification. Immediately after solubilization the internal standard consisting of 100 ng/mL d_3_-imatinib or d_6_-nilotinib was added. Protein concentrations were determined using the Bio-Rad protein assay dye reagent concentrate. Three independent experiments were performed in triplicate. Data are presented relative to the control group, which is indicated by the dashed line in Fig. 1. Average absolute uptake values of the different experiments are presented in Supplementary Table 1. Conditions showing a marked reduction in intracellular TKI concentration were further evaluated by exposing the cells to 1 µM of the TKI combined with 5 µM elacridar for P-gp inhibition and 1 µM KO143 for BCRP inhibition, data is shown as a percentage of the intracellular concentration under control values. In most conditions intracellular TKI concentrations were higher in inhibitor-treated cells compared to untreated conditions, suggesting that the inhibitors also blocked endogenously expressed HEK293 efflux transporters. Average control uptake and SEM values are shown in supplemental Table 1.

### LC/MS-MS analysis

Imatinib, nilotinib, dasatinib, bosutinib, ponatinib, and asciminib concentrations were measured in cell lysates with the use of liquid chromatography-tandem mass spectrometry (LC-MS/MS) analysis using an Acquity UPLC (Waters, Milford, MA, United States) equipped with a C18 UPLC column (Acquity HSST3 C18, 2.1 × 100 mm, Waters, Milford, MA, United States) coupled to a Xevo TQ-S (Waters) triple quadrupole mass spectrometer. Instrument settings for quantifying TKI concentrations were optimized in-house; details are provided in the supplementary section.

### Preparation of membrane vesicles

Membrane vesicles overexpressing enhanced Yellow Fluorescent Protein (eYFP, control), P-glycoprotein (P-gp), or Breast Cancer Resistance Protein (BCRP) were obtained from PharmTox (www.pharmtox.nl) Nijmegen, the Netherlands) and produced as previously described [20].

### TKI-dependent inhibition of P-gp and BCRP transport activity

Uptake of 3.6 nM/7.5 nCi or 7.2 nM/15 nCi [^3^H]-NMQ and 30.2 nM/50 nCi [^3^H]-ES into P-gp and BCRP overexpressing inside-out membrane vesicles, respectively, was determined as described before [21–25]. For the first P-gp vesicle experiments using imatinib, bosutinib and ponatinib 7.2 nM/15 nCi [^3^H]-NMQ was used, but concentrations were later reduced to minimize background signal. Concentrations of imatinib, nilotinib, dasatinib, bosutinib, ponatinib and asciminib that were added to the reaction mixture are indicated in the figure. Three separate experiments were performed in triplicate.

### Data analysis

Data from cellular experiments were expressed as means ± standard error of the means (SEM) from three independent experiments. Data from vesicular assays were presented as means ± standard deviation (SD). IC_50_ values for BCRP and P-gp were determined by fitting the concentration-inhibition data to a log(inhibitor) vs. response model by nonlinear regression analysis using GraphPad Prism version 9.5.0 (GraphPad Software Inc, San Diego, California). The bottom was constrained to > 0 and average Hill slopes were between − 0.79 and − 1.54. Assuming competitive inhibition, values for the inhibition constant (K_i_) were calculated from the obtained IC_50_ values using the Cheng-Prusoff Eq. [26]. The Michaelis-Menten constant (mean and SEM) for [^3^H]-ES in our BCRP vesicle preparation and [^3^H]-NMQ in P-gp vesicles was previously determined to be 6.1 µM (4.5–8.2 µM) and 2.2 µM (1.7–2.7 µM), respectively [27, 28]. Given the low substrate concentrations, IC_50_ and K_i_ values can be considered identical. Differences in intracellular TKI concentrations between the control and the transporter of interest were statistically analyzed according to a one-sample t-test compared to 100%. Significance was indicated as *P ≤ 0.05, **P ≤ 0.01, ***P ≤ 0.001, or ‘ns’ for non-significant results.

## Results

The potential of P-gp and BCRP to transport asciminib, imatinib, nilotinib, dasatinib, bosutinib and ponatinib was assessed by measuring the intracellular drug concentration in HEK293 cells overexpressing the transporter of interest (Fig. 1a). When exposed to 1 µM of the TKI, P-gp clearly effluxes asciminib (*p* = 0.113), imatinib (*p* = 0.0038), and dasatinib (*p* = 0.0004), and to a smaller extent bosutinib (*p* = 0.0426), as compared to mock transduced control cells. When exposed to 0.1 µM of the TKI, the intracellular imatinib concentration in P-gp overexpressing cells was significantly reduced (*p* = 0.0138), whereas intracellular reductions of asciminib, dasatinib and bosutinib of 16.7%, 9.6% and 9.7%, respectively, did not reach statistical significance. In BCRP-overexpressing HEK293 cells, a significant reduction in intracellular concentrations of asciminib (*p* = 0.0036), imatinib (*p* = 0.0192), and dasatinib (*p* = 0.0134) were observed when cells were exposed to 1 µM of the TKI. When BCRP-overexpressing cells were exposed to 0.1 µM TKI, intracellular concentrations of asciminib (*p* = 0.0060) and imatinib (*p* = 0.0021) were significantly reduced. Exposing transporter-overexpressing cells to a 10-fold reduced TKI concentration resulted in the same trend for all TKI-transporter combinations, except for asciminib and dasatinib in combination with P-gp. For these two combinations, no P‑gp–mediated transport was observed, which is consistent with their high Ki values of 30 µM or higher, which can be interpreted as Km values under the assumption of competitive inhibition. At an exposure of 0.1 µM, the substrate concentration is far below the Km of P‑gp, resulting in efflux levels too low to be detected. To confirm the transporter specificity of the TKIs that showed the most notable transporter-mediated reduction in cellular concentrations, i.e. asciminib, dasatinib, and imatinib, the inhibitors elacridar and KO143 were used to block P-gp and BCRP, respectively (Fig. 1b). Their intracellular concentrations in P-gp- and BCRP-transduced cells could be restored to at least control levels, indicating transporter inhibition, although for imatinib P-gp the difference did not reach statistical significance.

**Fig. 2:**
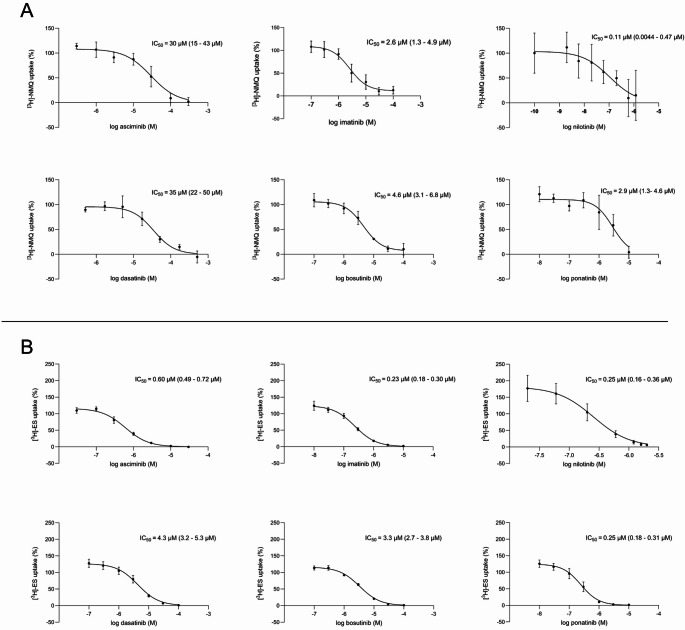
Inhibition of P-gp-mediated [^3^H]-NMQ (**A**) and BCRP-mediated [^3^H]-ES (**B**) transport by asciminib, imatinib, nilotinib, dasatinib, bosutinib and ponatinib is expressed as percentage of the control. Corresponding IC_50_ values with confidence intervals (CI) are displayed in the graph. Three separate experiments were performed in triplicate. Data are presented as mean ± SD.

The potential inhibitory effects of TKIs on P-gp and BCRP transport activity was investigated using membrane vesicles isolated from HEK293 cells overexpressing the respective transporters. It was shown that all TKIs are inhibitors of P-gp-mediated [^3^H]-NMQ transport (Fig. 2a) and BCRP-mediated [^3^H]-ES transport (Fig. 2b) in a concentration-dependent manner.

For P-gp, asciminib appeared to be a weak inhibitor, comparable to dasatinib, with both showing relatively high IC_50_ values. Nilotinib remained the most potent P-gp inhibitor, with an IC_50_ that is 270-times lower compared to asciminib. Conversely, asciminib demonstrated substantial potency against BCRP with an IC_50_ value of 0.60 µM, ranking it among the strong BCRP inhibitors imatinib, nilotinib and ponatinib. Dasatinib and bosutinib were 7 and 5.5 times less effective as BCRP inhibitors compared to asciminib, respectively. For most TKI and transporter combinations the maximum unbound concentration in plasma (C_max, u_) is lower compared to the inhibitory constant (K_i_), suggesting that inhibition of the transporter under therapeutic conditions is unlikely (Table 1). Some C_max, u_ values approach the K_i_, suggesting that asciminib and imatinib both hold the potential to inhibit BCRP.

**Table 1:** Overview of K_i_ and C_max_ values of TKIs for P-gp-mediated [^3^H]-NMQ transport and BCRP-mediated [^3^H]-ES transport activity

	*P*-gp K_i_ (µM)	BCRP K_i_ (µM)	Plasma protein binding	C_max_ (µM)	C_max, u_^7^ (µM)
asciminib	30	0.60	97% [29]	12.5^1^ [30]	0.38
imatinib	2.6	0.23	92% [31]	9.0^2^ [32]	0.72
nilotinib	0.11	0.25	> 97.5% [33]	3.1^3^ [34]	< 0.078
dasatinib	35	4.3	94% [35]	0.16^4^ [36]	0.0096
bosutinib	4.6	3.3	94% [37]	0.5^5^ [38]	0.031
ponatinib	2.9	0.25	> 99% [39]	0.14^6^ [40]	< 0.0014

^*1*^Mean steady-state at 200 mg BID. ^*2*^Steady-state with 1000 mg BID (twice daily) dosing regimen. ^*3*^Steady-state with 400 mg BID dosing regimen. ^*4*^Steady-state with 140 mg once daily dosing regimen. ^*5*^Multiple dose 500 mg dosing regimen. ^*6*^Mean steady-state after 45 mg dosing regimen. ^*7*^C_max, u_ is the unbound drug at C_max_; calculated based on the given plasma protein binding and C_max._

## Discussion

P-gp and BCRP are drug transporting proteins that are widely distributed throughout the human body. Because of their well-established roles in drug efflux, they are often studied in relation to treatment intolerance or treatment failure, including TKIs used in the treatment of CML. The novel STAMP inhibitor asciminib is less well characterized in this regard, and conflicting data exist for the role of P-gp in nilotinib and bosutinib efflux. We therefore aimed to study the interactions of asciminib with P-gp and BCRP. Other TKIs approved for CML were included to clarify previously ambiguous interactions and to provide a comprehensive overview with our in vitro approach. Asciminib, but also imatinib and dasatinib have previously been shown to be substrates for P-gp, consistent with the data presented here [10, 41–44]. Divergent results have been published regarding the interaction of nilotinib and P-gp. Hegedus et al. [10] reported that P-gp overexpression did not reduce intracellular nilotinib concentrations, consistent with findings of Davies et al. [11], who concluded that nilotinib was not transported by P-gp. On the other hand, Mahon et al. [13] and Dohse et al. [12] demonstrated a protective effect of P-gp overexpression in cells exposed to nilotinib. Moreover, Shukla et al. [14] provided evidence for P-gp mediated efflux of both a fluorescent nilotinib derivative and radio-labelled nilotinib. In the present study P-gp-mediated nilotinib efflux was not observed. Differences may be attributed to the model systems employed; for example Shukla et al. used transwell assays and isolated rat brain capillaries [14]. In the studies performed by Mahon et al. [13] and Dose et al. [12], cell survival was assessed as the primary endpoint, resulting in a more indirect readout and requiring a longer incubation time with nilotinib. Regarding the interaction between P-gp and bosutinib conflicting reports can be found. Transporter overexpression has shown to prevent cellular incorporation of C-14 radiolabeled bosutinib [16]. In contrast, the intracellular bosutinib concentration did not differ significantly between K562 cells overexpressing P-gp and their parental controls [10]. In the present study, the bosutinib concentration in P-gp overexpressing cells was reduced only modestly, indicating that it is a poor P-gp substrate. Ponatinib has previously been shown not to be transported by P-gp, in agreement with the data presented in this study [10, 45]. Based on previously performed studies, it was hypothesized that asciminib, imatinib, nilotinib, and dasatinib, but not ponatinib and bosutinib, are substrates of BCRP [10, 43, 45, 46]. In the present study, asciminib indeed proved to be a substrate of BCRP, similar to imatinib and dasatinib. Discrepancies in the reported interactions of nilotinib may reflect differences in TKI concentrations, cell models and analytical methods [47]. Future studies using patient-derived cells or in vivo systems may help to resolve these inconsistencies. TKIs may also exhibit an inhibitory effect on P-gp and BCRP. Therefore, we determined K_i_ values to characterize the inhibitory potency of asciminib and the 5 other TKIs against P-gp and BCRP using transporter-overexpressing membrane vesicles. Asciminib has been reported to inhibit P-gp with a K_i_ value of 22 µM, which is broadly consistent with the present study, where a value of 30 µM was found [48]. As the unbound drug concentration in patients at C_max_ is 0.38 µM, the influence of asciminib-mediated P-gp inhibition is likely negligible. Previous in vitro studies have shown that imatinib and nilotinib inhibit P-gp-mediated dasatinib transport with IC_50_ values of 2.4 and 6.1 µM, respectively (43). Nilotinib was also shown to increase the sensitivity of P-gp-overexpressing cells to colchicine, vinblastine, and paclitaxel [49, 50]. As for dasatinib, it was reported that at therapeutically relevant concentrations no inhibition of P-gp mediated substrate efflux was seen [51]. Bosutinib inhibited P-gp only at concentrations exceeding 1 µM levels that are supratherapeutic and clinically irrelevant [10]. Ponatinib has been reported to enhance substrate uptake in cells overexpressing P-gp and BCRP, indicating transporter inhibition [52]. In the present work all TKIs inhibited P-gp in a concentration-dependent matter, with the inhibitory potency ranking from nilotinib> imatinib> ponatinib> bosutinib> asciminib> dasatinib (strong to weak), grossly in accordance with previous research. Compared to other P-gp inhibitors that have previously been studied in the same model system, asciminib has a similar inhibitory potency as proscillaridin A and rifampicin, for which an IC_50_ values of 25 µM and 29 µM respectively have been reported [21, 28]. It has been described that asciminib inhibits BCRP-mediated transport with a K_i_ value of 24 µM, while in the present study a value of 0.60 µM was found [48]. This might be attributable to differences in the model systems used or the choice of the substrate, which is not disclosed in the public assessment reports of the European Medicines Agency (EMA) [48]. The K_i_ value found for asciminib in the present study is in the same order of magnitude compared to the unbound fraction at C_max_, suggesting a potential for interference with concomitantly administered substrate drugs. It has previously been described that imatinib and nilotinib reduced BCRP-mediated efflux of Hoechst 33,342 dye in a dose-dependent and reversible manner [53, 54], and both inhibited BCRP-mediated dasatinib transport with IC_50_ values of 0.94 and 2.5 µM, respectively [43]. Nilotinib also increased the sensitivity of BCRP-overexpressing cells to mitoxantrone and doxorubicin [49]. The inhibitory potential of bosutinib on BCRP remains uncertain. Although bosutinib has been reported as a BCRP inhibitor, in vitro studies indicate only limited inhibition at clinically relevant concentrations [55–57]. Dasatinib has been shown to inhibit BCRP at high, supratherapeutic concentrations [10, 12]. For ponatinib, BCRP inhibition has been reported although K_i_ values have not been determined [58]. In the current study all TKIs inhibited BCRP in a concentration-dependent manner, with inhibitory potencies ranked as imatinib> nilotinib/ponatinib> asciminib> bosutinib> dasatinib (strong to weak). These results are largely in agreement with previously published results. Compared to other BCRP inhibitors that have previously been studied in the same model system, asciminib proved to be five times more potent than clofazimine, which inhibited [^3^H]-ES with an IC_50_ of 3.2 µM [21]. Notably, BCRP transporter activity was slightly stimulated at low TKI concentrations, suggesting a possible allosteric interaction that is unlikely to have a meaningful impact in vivo [59, 60]. All CML-targeted TKIs are inhibitors of both P-gp and BCRP in a concentration-dependent manner. For most TKI-transporter combinations, K_i_ values are well above the C_max, u_, suggesting limited clinical relevance of drug-mediated transporter inhibition. However, the inhibitory potency of asciminib against BCRP, as well that of imatinib and nilotinib against both P-gp and BCRP, warrants further investigation into potential drug-drug interactions.

In conclusion, this study highlights asciminib as a P-gp and BCRP substrate and inhibitor, offering new insight into its transporter interactions. It also clarifies the roles of P-gp in the transport of nilotinib and to a more modest extent for bosutinib, contributing to a better understanding of TKI pharmacokinetics and potential resistance mechanisms.

## Supplementary Information

Below is the link to the electronic supplementary material.

Supplementary Material 1

## Data Availability

The authors declare that all the data supporting the findings of this study are contained within the paper.
