# Supplementary material for: Characterizing P-glycoprotein and Breast Cancer Resistance Protein interactions of asciminib among other tyrosine kinase inhibitors used in chronic myeloid leukemia
Source: Cancer Chemother Pharmacol. 2026 May 12;96(1):47. doi: 10.1007/s00280-026-04891-z (PMC13167888; doi:10.1007/s00280-026-04891-z)
Supplement: Supplementary file 1 — Supplementary Material 1 [file 280_2026_4891_MOESM1_ESM.docx]

Supplemental file 1

*Detailed method of LC/MS-MS based TKI detection*

Intracellular imatinib, nilotinib, dasatinib, bosutinib, ponatinib, and asciminib concentrations were measured in cell lysate with the use of liquid chromatography-tandem mass spectrometry (LC–MS/MS)  analysis using an Acquity UPLC (Waters, Milford, MA, USA) equipped with a C18 UPLC column (Acquity HSST3 C18, 2.1 × 100 mm, Milford, MA, USA, Waters) coupled to a Xevo TQ-S (Waters) triple quadrupole mass spectrometer. Column temperature was set to 40°C and tray temperature was 15°C. Following subsequent centrifugation (13,000g for 3 min), 1μL of the protein-free clear supernatant was injected into the LC–MS to quantify the six TKIs. Solvent A consisted of 1mM NH4FA + 0,1% formic acid in H2O and solvent B consisted of 0,1% formic acid in MeOH. Separation was performed at a flow rate of 350 µl/min under the following gradient conditions: 0 min 70% eluent A, 2.0 min 0% eluent A, 3.0 min 0% eluent A, 4.0 min 70% eluent A. The effluent from the UPLC was passed directly into the electrospray ion source. Positive electrospray ionization was achieved using nitrogen as a desolvation gas with ionization voltage at 600 V. The source temperature was set at 500 °C and argon was used as collision gas, in line with previously used protocols [55]. The following MRM transitions were used: m/z 494.2 (parent ion) to m/z 394.2 and m/z 217.1 (both product ions) for imatinib, m/z 530.1 (parent ion) to m/z 289.1 and m/z 259.3 (both product ions) for nilotinib, m/z 488.1 (parent ion) to m/z 401.1 and m/z 232.1 (both product ions) for dasatinib, m/z 530.1 (parent ion) to m/z 141.0 and m/z 69.9 (both product ions) for bosutinib, m/z 533.2 (parent ion) to m/z 260.0 and m/z 100.9 (both product ions) for ponatinib, m/z 450.1 (parent ion) to m/z 432.1 and m/z 239.1 (both product ions) for asciminib, m/z 497.2 (parent ion) to m/z 394.2 (product ion) for d3-imatinib, and m/z 536.2 (parent ion) to m/z 295.1 (product ion) for d6-nilotinib. Calibration curves went up to 100ng/ml (asciminib and dasatinib), 200ng/ml (ponatinib and imatinib), 300ng/ml (nilotinib), or 400ng/ml (bosutinib).

Supplementary Table 1

*Absolute uptake values (pmol/mg protein) of TKIs exposed to HEK293 control cells (EYFP transduced) under different experimental conditions. Results are presented as mean* ± *SEM*

| Condition | asciminib | imatinib | nilotinib | dasatinib | bosutinib | ponatinib |
| --- | --- | --- | --- | --- | --- | --- |
| 1 µM TKI | 170 ± 20 (n=10) | 160 ± 20 (n=6) | 120 ± 30 (n=3) | 190 ± 20 (n=10) | 520 ± 80 (n=4) | 570 ± 140 (n=4) |
| 0,1 µM TKI | 19 ± 3  (n=3) | 12 ± 1  (n=3) | 13 ± 3  (n=3) | 10 ± 2  (n=3) | 24 ± 2  (n=3) | 30 ± 10  (n=3) |
| 1 µM TKI + elacridar^a^ | 220 ± 20 (n=3) | 130 ± 10 (n=3) |  | 210 ± 50 (n=3) |  |  |
| 1 µM TKI + KO143^b^ | 350 ± 50 (n=3) | 130 ± 7  (n=3) |  | 320 ± 80 (n=3) |  |  |

^a^P-gp inhibitor, ^b^BCRP inhibitor
